# The wedge insole for the treatment of knee osteoarthritis: A systematic review protocol

**DOI:** 10.1097/MD.0000000000017168

**Published:** 2019-09-13

**Authors:** Shuling Chen, Yicheng Sun, Guanhua Ma, Xunlu Yin, Long Liang

**Affiliations:** a Jinan Zhangqiu District Hospital of Traditional Chinese Medicine, Shandong Province; b Wangjing Hospital of Chinese Academy of Chinese Medical Sciences, Beijing, China.

**Keywords:** knee osteoarthritis, protocol, systematic review, wedge insole

## Abstract

**Background:**

Functional limitations and pain are common presenting complaints for people suffering from knee osteoarthritis. Wedge insole can be sued for treatment of knee osteoarthritis. Hence, we conducted a systematic review and meta-analysis to explicit the efficacy of wedge insole in the treatment of knee osteoarthritis.

**Methods:**

A systematic literature search for studies will be performed in MEDLINE, Embase, the Chinese National Knowledge Infrastructure Database (CNKI), Cochrane Library, Web of Science. The methodological quality of the included studies using the risk bias assessment tool of Cochrane. Funnel plot will be used to assess the reporting bias. And the level of evidence for results are assessed by the GRADE method. Statistical analysis is conducted with Revman 5.3.

**Results:**

This systematic review and meta-analysis will provide a synthesis of evidences for wedge insole on knee osteoarthritis.

**Conclusion:**

The conclusion of this study will provide recommendations to assess effectiveness of exercise on knee osteoarthritis, which may further guide clinical practice.

**PROSPERO registration number:**

CRD42018096804

## 1 Introduction

Knee osteoarthritis, as a leading cause of disability, is a prevalent joint disease is associate to acute or chronic injures from normal wear and tear, age, obesity, etc.^[1,2]^ Knee osteoarthritis is an extreme burden on society, and this burden will increase with time goes on.^[3]^ An estimated 13 million adults aged 60 years and older in US have radiographic knee osteoarthritis, with approximately 4 million of those having symptomatic manifestations.^[1,3]^ Besides, treating lower extremity posttraumatic osteoarthritis, which only accounts for approximately 10% of all cases of knee osteoarthritis, cost $11.79 billion in 2005. And the cost is increasing every year annually.^[4]^ At present, the treatment of knee osteoarthritis is divided into surgical and conservative methods. However, surgical treatments are generally considered only when knee osteoarthritis reaches its advanced stage.^[5]^ Hence, nonoperative treatments are often useful for patients with “early” stages (Kellgren and Lawrence Grades 1–3) of knee osteoarthritis.^[6]^ Furthermore, Hunter study revealed the recommended hierarchy of treatment measures should consist of nonpharmacologic methods first.^[7]^

The wedge insole, as a commonly conservative alternative, is a thicker wedge at the lateral part than other area placed under the sole, which forms an angle.^[8]^ Wedge insole may provide knee pain relief by improving the load distribution within the knee and the alignment of the knee.^[9,10]^ Also, Some clinical trials have demonstrated the role of wedge insole in the treatment of knee osteoarthritis.^[11–13]^ However, according to the study conducted by Bennell et al, it cannot significantly improve the symptoms of knee osteoarthritis.^[14]^ In addition, some systematic review and meta-analyses, which is a higher level of evidence-based research, have drawn different conclusions.^[8,15,16]^ These confusing outcomes have seriously hampered clinical decision-making for orthopedist. Accordingly, there is an urgent need for a meta-analysis of the most comprehensive and appropriate methodological to evaluate the effect of wedge insole on knee osteoarthritis in order to guide the clinical practice of knee osteoarthritis.

## 2 Methods

This is the second study of literature, so no ethical approval and patient consent is required. The protocol of this meta-analysis has been registered on the

International Prospective Register of Systematic Reviews (PROSPERO) (registration no. CRD42018096804) basing on the Preferred Reporting Items for Systematic Reviews and Meta-Analyses Protocols (PRISMA-P) statement guidelines.^[17]^

### 2.1 Literature research

We will search the following electronic bibliographic databases: MEDLINE, Embase, the Chinese National Knowledge Infrastructure Database (CNKI), Cochrane Library, Web of Science. No limits are imposed on study date or publication language, type, and status. Search strategies will be developed from keywords indexed in the Medical Subject Headings (MeSH) and random words, which are related to wedge insole and knee osteoarthritis. The search keywords used include the following: “Knee Osteoarthritis”, “Osteoarthritis of the knee”, “Knee Osteoarthritides”, “Osteoarthritis of Knee”, “Osteoarthritis of Knees”, “Wedge Insole”, “Foot Orthosis”, “Foot Orthotic Device”, “Foot Support”, “ Arch Support”, “Foot Arch”, “Orthotic Shoe Inserts”, “Orthotic Insoles”, “Insoles, Orthotic”, and “Orthotic Insole”.

### 2.2 Inclusion criteria

The retrieved literature is screened by 2 independent reviewers to evaluate eligibility, and any discrepancies are settled by discussion. First, the titles and abstracts of searched studies are screened. Then, full papers are reviewed according to the following reference criteria:

(1) randomized controlled trial;
(2) type of participants must be patients with knee osteoarthritis;
(3) experimental studies using wedge insole.
(4) control group is not restricted, but the treatment method should not include wedge insole.

When multiple time points were reported either in one particular report of a study or over the course of several articles from the same study, the longest follow-up period on treatment is considered in our article.

### 2.3 Data extraction

The following data are independently extracted by 2 authors via a pre-designed form: the name of first author, year of publication, country, sample size, age, gender of patients, disease course, follow-up duration, outcome and intervention period. We will contact the authors by email or in other ways if the data are missing, wrong, or unclear. The authors resolve any disagreements by discussion, including input from a third author if required.

### 2.4 Quality assessment

We assessed the risk of bias of RCTs in this review using the Cochrane Collaboration Risk of Bias Tool.^[15]^ And risk of bias is assessed according to the Risk of Bias Tool for Randomized Clinical Trials provided by Cochrane Collaborarion Network. For included study, types of bias are divided into 3 levels: low, unclear, high. Two authors independently assess the risk of bias of the included studies. The divergences between the two review authors will be resolved by consensus, including input a third independent review author if required.

### 2.5 Outcome measures

The primary outcome is visual analogue scale (VAS), and the secondary outcome are The Western Ontario and McMaster Universities Osteoarthritis Index (WOMAC). VAS was used to evaluate the pain degree of knee osteoarthritis, and the WOMAC index reflects the functional status of knee osteoarthritis.

### 2.6 Data synthesis and statistical analysis

The dichotomous data is expressed as the risk ratio (RR) with 95% confidence intervals (CI). And mean difference (MD) with 95% confidence intervals (CI) is used to assess the difference in the continuous outcomes. Statistical heterogeneity across the included studies will be examined using the I^2^ statistic, with an I^2^ > 50% indicate the possibility level of heterogeneity, resulting in the selection of a random-effects model for merging of data. Otherwise, no obvious heterogeneity will be considered to be present in the included studies for values of I^2^ < 50%, in which case the fixed-effects model will be chosen. When the heterogeneity of the merged results across groups is large, sensitivity analysis can be considered to analyze the robustness of the results, and the method of excluding articles one by one is adopted. Funnel plot will be used to assess the reporting bias. Data regarding outcomes in the eligible trials are combined using the RevMan 5.3 software, and the significance threshold will be a 2-sided *P* < .05.

### 2.7 GRADE

The quality of the evidence can be assessed by the Grading of Recommendations Assessment, Development, and Evaluation (GRADE) method. The evaluation system of each index is divided into four levels: high, moderate, low and very low.

## 3 Discussion

Use of the wedge insole, as is a nonoperative approach, is used for the treatment of knee osteoarthritis in clinical practice, because it can decrease the loading stress of knee compartment.^[18]^ Besides, the wedge insole can significantly increase femorotibial angle, which means reduce the knee varus deformity.^[8]^ However, Maly et al's study have shown no association with between severity of pain and knee load.^[19]^ And even the opposite one in other study.^[20]^ So, it is not clear whether the use of wedge insole can improve the pain and other symptoms of knee osteoarthritis. In addition, the confusing results of many studies on this topic also hinder the use of insoles in the treatment of knee osteoarthritis.^[9,14,15,16]^

It is, therefore, necessary to carry out a study to assess the efficacy of wedge insole therapy for knee osteoarthritis. Furthermore, we hope the results of this study can help to form the clinical recommendation for knee osteoarthritis and to provide more high-level evidence about the application of wedge insole.

## Author contributions

**Conceptualization:** Yicheng Sun.

**Data curation:** Shuling Chen, Yicheng Sun, Guanhua Ma.

**Methodology:** Shuling Chen, Yicheng Sun, Xunlu Yin.

**Resources:** Shuling Chen, Guanhua Ma, Long Liang.

**Software:** Shuling Chen, Yicheng Sun, Xunlu Yin, Long Liang.

**Supervision:** Yicheng Sun.

**Writing – original draft:** Shuling Chen, Yicheng Sun.

**Writing – review & editing:** Shuling Chen, Yicheng Sun, Guanhua Ma.

## References

[1] Thomas AC Hubbard-Turner T Wikstrom EA Epidemiology of posttraumatic osteoarthritis. J Athl Train 2017;52:491–6. 27145096 10.4085/1062-6050-51.5.08 PMC5488839

[2] Dor A Kalichman L A myofascial component of pain in knee osteoarthritis. J Bodyw Mov Ther 2017;21:642–7. 28750978 10.1016/j.jbmt.2017.03.025

[3] Sliepen M Brandes M Rosenbaum D Current physical activity monitors in hip and knee osteoarthritis: a review. Arthritis Care Res 2017;69:1460–6. 10.1002/acr.23170 PMC5656924 27998033

[4] Brown TD Johnston RC Saltzman CL Posttraumatic osteoarthritis: a first estimate of incidence, prevalence, and burden of disease. J Orthop Trauma 2006;20:739–44. 17106388 10.1097/01.bot.0000246468.80635.ef

[5] Vaishya R Pariyo GB Agarwal AK Non-operative management of osteoarthritis of the knee joint. J Clin Orthop Trauma 2016;7:170–6. 27489412 10.1016/j.jcot.2016.05.005 PMC4949406

[6] Lespasio MJ Piuzzi NS Husni ME Knee osteoarthritis: a primer. Perm J 2017;21:16–83. 10.7812/TPP/16-183 PMC5638628 29035179

[7] Hunter DJ Viscosupplementation for osteoarthritis of the knee. N Engl J Med 2015;372:1040–7. 25760356 10.1056/NEJMct1215534

[8] Zhang B Yu X Liang L Is the wedged insole an effective treatment option when compared with a flat (placebo) insole: a systematic review and meta-analysis. Evid Based Complement Alternat Med 2018;2018:8654107. 30622616 10.1155/2018/8654107 PMC6304499

[9] Schmalz T Knopf E Drewitz H Analysis of biomechanical effectiveness of valgus-inducing knee brace for osteoarthritis of knee. J Rehabil Res Dev 2010;47:419–29. 20803386 10.1682/jrrd.2009.05.0067

[10] Khosravi M Arazpour M Sharafat Vaziri A An evaluation of the use of a lateral wedged insole and a valgus knee brace in combination in subjects with medial compartment knee osteoarthritis (OA). Assist Technol 2019;31:1–8. 30945994 10.1080/10400435.2019.1595788

[11] Rodrigues PT Ferreira AF Pereira RM Effectiveness of medial-wedge insole treatment for valgus knee osteoarthritis. Arthritis Rheum 2008;59:603–8. 18438931 10.1002/art.23560

[12] Pham T Maillefert JF Hudry C Laterally elevated wedged insoles in the treatment of medial knee osteoarthritis. A two-year prospective randomized controlled study. Osteoarthritis Cartilage 2004;12:46–55. 14697682 10.1016/j.joca.2003.08.011

[13] Maillefert JF Hudry C Baron G Laterally elevated wedged insoles in the treatment of medial knee osteoarthritis: a prospective randomized controlled study. Osteoarthritis Cartilage 2001;9:738–45. 11795993 10.1053/joca.2001.0470

[14] Bennell KL Bowles KA Payne C Lateral wedge insoles for medial knee osteoarthritis: 12 month randomised controlled trial. BMJ 2011;342:d2912. 21593096 10.1136/bmj.d2912 PMC3100910

[15] Shaw KE Charlton JM Perry CKL The effects of shoe-worn insoles on gait biomechanics in people with knee osteoarthritis: a systematic review and meta-analysis. Br J Sports Med 2018;52:238–53. 28684391 10.1136/bjsports-2016-097108

[16] Xing F Lu B Kuang MJ A systematic review and meta-analysis into the effect of lateral wedge arch support insoles for reducing knee joint load in patients with medial knee osteoarthritis. Medicine 2017;96:e7168. 28614253 10.1097/MD.0000000000007168 PMC5478338

[17] Shamseer L Moher D Clarke M Preferred reporting items for systematic review and meta-analysis protocols (PRISMA-P) 2015: elaboration and explanation. BMJ 2015;350:g7647 doi: 10.1136/bmj.g7647. 25555855 10.1136/bmj.g7647

[18] Ishii Y Deie M Fujita N Effects of lateral wedge insole application on medial compartment knee osteoarthritis severity evaluated by ultrasound. Knee 2017;24:1408–13. 28970118 10.1016/j.knee.2017.09.001

[19] Maly MR Costigan PA Olney SJ Mechanical factors relate to pain in knee osteoarthritis. Clin Biomech (Bristol, Avon) 2008;23:796–805. 10.1016/j.clinbiomech.2008.01.014 18346827

[20] Briem K Axe MJ Snyder-Mackler L Medial knee joint loading increases in those who respond to hyaluronan injection for medial knee osteoarthritis. J Orthop Res 2009;27:1420–5. 19408285 10.1002/jor.20899

